# Sleep Disordered Breathing in Children with Autism Spectrum Disorder: An In-Depth Review of Correlations and Complexities

**DOI:** 10.3390/children10101609

**Published:** 2023-09-27

**Authors:** Marco Zaffanello, Giorgio Piacentini, Luana Nosetti, Leonardo Zoccante

**Affiliations:** 1 Department of Surgery, Dentistry, Paediatrics and Gynaecology, University of Verona, 37126 Verona, Italy; giorgio.piacentini@univr.it; 2 Department of Pediatrics, Pediatric Sleep Disorders Center, “F. Del Ponte” Hospital, Insubria University, 21100 Varese, Italy; luana.nosetti@uninsubria.it; 3 Child and Adolescent Neuropsychiatry Unit, Maternal-Child Integrated Care Department, Integrated University Hospital Verona, 37126 Verona, Italy; leonardo.zoccante@aovr.veneto.it

**Keywords:** autism spectrum disorder, children, sleep apnea, sleep-disordered breathing, sleep quality

## Abstract

Sleep-disordered breathing is a significant problem affecting the pediatric population. These conditions can affect sleep quality and children’s overall health and well-being. Difficulties in social interaction, communication, and repetitive behavioral patterns characterize autism spectrum disorder. Sleep disturbances are common in children with ASD. This literature review aims to gather and analyze available studies on the relationship between SDB and children with autism spectrum disorder. We comprehensively searched the literature using major search engines (PubMed, Scopus, and Web of Science). After removing duplicates, we extracted a total of 96 records. We selected 19 studies for inclusion after a thorough title and abstract screening process. Seven articles were ultimately incorporated into this analysis. The research findings presented herein emphasize the substantial influence of sleep-disordered breathing on pediatric individuals diagnosed with autism spectrum disorder (ASD). These findings reveal a high incidence of SDB in children with ASD, emphasizing the importance of early diagnosis and specialized treatment. Obesity in this population further complicates matters, requiring focused weight management strategies. Surgical interventions, such as adenotonsillectomy, have shown promise in improving behavioral issues in children with ASD affected by OSA, regardless of their obesity status. However, more comprehensive studies are necessary to investigate the benefits of A&T treatment, specifically in children with ASD and OSA. The complex relationship between ASD, SDB, and other factors, such as joint hypermobility and muscle hypotonia, suggests a need for multidisciplinary treatment approaches. Physiotherapy can play a critical role in addressing these intricate health issues. Early sleep assessments and tailored weight management strategies are essential for timely diagnosis and intervention in children with ASD. Policy initiatives should support these efforts to enhance the overall well-being of this population. Further research is crucial to understand the complex causes of sleep disturbances in children with ASD and to develop effective interventions considering the multifaceted nature of these conditions.

## 1. Introduction

Sleep-disordered breathing (SDB) represents a significant issue affecting the pediatric population [1,2]. Within the broader pediatric population, obstructive sleep apnea (OSA) ranges from 2% to 5%, although in specific medical contexts, its prevalence can be significantly higher [3]. These conditions can have severe consequences on the health and well-being of children [1,4,5].

In the broader context, independent risk factors for OSA encompass persistent snoring for ≥3 months, tonsillar and adenoid hypertrophy, and obesity (Xu et al., 2020). Moreover, frequent respiratory infections [6] may amplify the impact, particularly when coupled with factors such as tonsillar and adenoid hypertrophy or obesity. Muscle hypotonia in children with genetic comorbidities [7,8,9] may serve as concurrent catalysts exacerbating SDB. The intricate interplay of SDB can orchestrate disruptions in sleep patterns and intermittent hypoxia, exerting a discernible impact on the cognitive and behavioral faculties of children [10,11,12]. Consequently, delving into the long-term trajectories of these disorders and maintaining vigilant follow-up mechanisms becomes imperative [1,13].

Neurodevelopmental disorders constitute a wide-ranging category of medical conditions that profoundly impact the intricate development of the nervous system, particularly during the critical phases of brain maturation. The delicate interplay of factors shaping proper nervous system development intertwines with the multifaceted complexities characterizing the manifestation of autism spectrum disorder (ASD) during the crucial stages of childhood or infancy [14,15,16], exploring the nuanced aspects of the condition. The resulting impact reverberates across a comprehensive spectrum of domains, encompassing the intricate threads of communication, the fabric of learning, the dance of social behavior, and the tapestry of motor skills. While ASD maintains its distinct identity with unique attributes, acknowledging the latent potential for multiple neurodevelopmental disorders to converge within the presentation of children remains of paramount significance [17,18,19].

### Aims of the Study

This study seeks to investigate the existing comprehension of the complex association between ASD and SDB in the pediatric population, the underlying mechanisms involved, and the significance of timely identification and intervention for SDB in children with ASD. The study seeks to illuminate how early interventions can enhance sleep quality, address behavioral challenges, and improve the overall well-being of affected children.

## 2. Materials and Methods

We conducted a literature search using major search engines (PubMed, Scopus, and Web of Science; access date 15 July 2023), employing the relevant keywords listed below:

PubMed: (“autism spectrum disorders” OR “autism” OR “autistic” OR “Asperger” OR “Pervasive developmental disorder”) AND (“sleep-disordered breathing” OR “sleep apnea” OR “sleep apnoea” OR “sleep disorders breathing”) AND (“Polysomnography” OR “Treatment outcomes” OR “Complications”) AND (“Children” OR “Pediatric patients” OR “infant”).

Scopus: (TITLE-ABS-KEY(“autism spectrum disorders”) OR TITLE-ABS-KEY(“autism”) OR TITLE-ABS-KEY(“autistic”) OR TITLE-ABS-KEY(“Asperger”) OR TITLE-ABS-KEY(“Pervasive developmental disorder”)) AND (TITLE-ABS-KEY(“sleep disordered breathing”) OR TITLE-ABS-KEY(“sleep apnea”) OR TITLE-ABS-KEY(“sleep apnoea”) OR TITLE-ABS-KEY(“sleep disorders breathing”)) AND (TITLE-ABS-KEY(“polysomnography”) OR TITLE-ABS-KEY(“treatment outcomes”) OR TITLE-ABS-KEY(“complications”)) AND (TITLE-ABS-KEY(“children”) OR TITLE-ABS-KEY(“pediatric”) OR TITLE-ABS-KEY(“infant”)).

Web of Science: TS = (“autism spectrum disorders” OR “autism” OR “Pervasive developmental disorder” OR “autistic” OR “Asperger”) AND TS = (“sleep-disordered breathing” OR “sleep apnea” OR “sleep apnoea” OR “sleep disorders breathing”) AND TS = (“Polysomnography” OR “Treatment outcomes” OR “Complications”) AND TS = (“Children” OR “Pediatric” OR “infant”).

### Inclusion and Exclusion Criteria

The inclusion criteria comprised the pediatric age group, autism, autistic, pervasive, sleep apnea, SDB, OSA, and polysomnography (PSG). Exclusion criteria encompassed non-English articles, reviews, case reports, case series, or letters, studies focusing on adults (>18 years), studies lacking specific outcome reporting, and duplicate studies (i.e., those published multiple times or identified from different data sources).

## 3. Results

**Figure 1:**
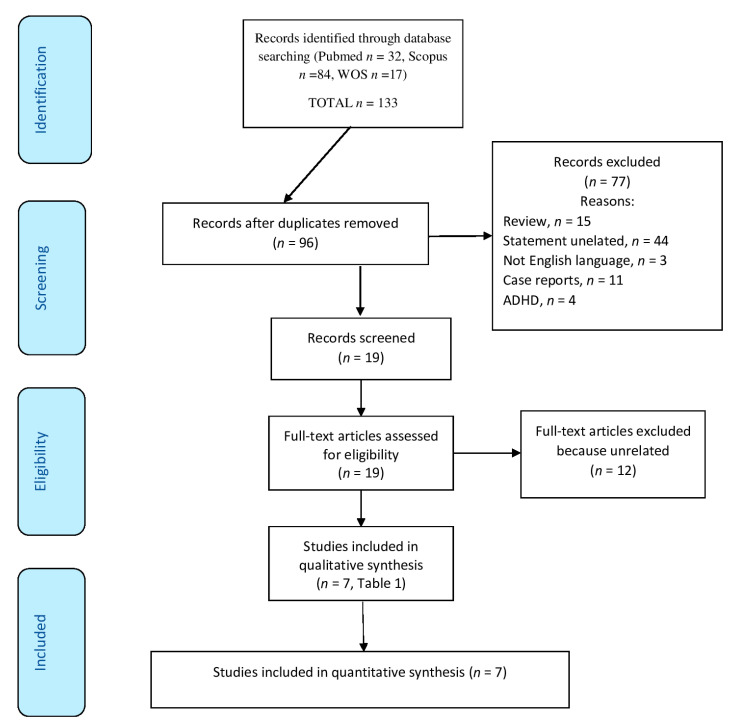
The PRISMA flow diagram graphically illustrates the study selection process, indicating the number of studies incorporated at each phase (export date: 5 June 2023).

Ninety-six records were initially identified, accounting for duplicates. Through a meticulous screening of titles and abstracts, we narrowed our selection to 19 studies that aligned with our research objectives. Subsequently, we comprehensively evaluated these studies to ascertain their relevance and quality. Following this procedure, we ultimately incorporated seven articles (Figure 1).

Four studies examined SDB within the broader context of sleep disorders in children with ASD (Table 1). Youssef et al. investigated the relationship between ferritin levels, fragmented sleep, and joint movements in children [20]. Tudor et al. explored the correlation between pain and sleep problems [21]. Elrod et al. delved into the risk of sleep disorders associated with diagnostic/surgical procedures in children [22]. Johnson et al. explored the psychometric properties of the Sleep Subscale of the Children’s Sleep Habits Questionnaire (CSHQ) [23]. Three studies (Table 1) specifically focused on OSA. Murata et al. examined behavioral changes following tonsillectomy and adenoidectomy interventions [24]. Tomkies et al. investigated predictors of OSA and severe OSA, along with demographic and clinical characteristics [25]. Additionally, a study compared OSA symptoms and the age of diagnosis [26].

Studies investigating the relationship between SDB and children with ASD have employed various methodologies and involved diverse subject groups. The survey conducted by Youssef et al. included a large population of children with ASD, analyzing participant data for ferritin levels and PSG data [20]. Tudor et al. evaluated parent-reported sleep habits using the CSHQ. They examined correlations between pain and sleep disorders, including SDB, to understand the impact of pain on sleep quality [21]. Elrod et al. analyzed ASD data from a broad sample of children, comparing it to a control group. They assessed the risk rates of sleep disorders and SDB. Johnson et al. evaluated sleep habits and SDB using the CSHQ and studied psychometric properties [23]. Murata et al. investigated OSA in children with and without ASD using PSG, assessing behavioral changes before and after treatment [24]. Tomkies et al. performed PSG on children, examining predictors of OSA and considering demographic and clinical variables [25]. Santapuram et al. reviewed clinical records, looking at diagnoses and treatments for SDB [26]. In summary, through diverse methodologies such as PSG analysis, the CSHQ, ICD-9 cm data, and clinical assessments, these studies have delved into the intricate relationship between SDB and ASD, revealing crucial aspects of this dynamic.

SDB represents a complex study area for children with ASD and has brought forth several significant findings. In the first study by Youssef et al., ferritin levels and BMI were not significantly correlated with OSA. This lack of association suggested that different factors might contribute to the onset of SDB in this population [20]. On the other hand, the investigation by Tudor et al. revealed that sleep behaviors and vocalizations could influence scores on the SDB subscale of the CSHQ. This finding suggests that SDB might be associated with specific vocal behaviors and overall sleep quality [21]. Elrod et al. uncovered a higher risk of developing sleep disorders, including OSA, in children with ASD [22]. These results underscore the need for a timely assessment and intervention to address such respiratory diseases. However, despite the association between ASD and SDB, loud snoring and other abnormal breathing behaviors were rarely found in the children examined in Johnson et al.’s study [23]. This suggests that while sleep disorders are essential to consider, they might vary significantly in frequency and presentation. The findings from Murata et al. highlighted the effect of OSA on the behaviors and overall well-being of children with ASD. OSA treatment emerged as a crucial element for improving behavioral issues [24]. Irrespective of obesity and age, the extent of the scope of OSA was further explored in the study by Tomkies et al., which revealed that OSA is common in children with ASD and can vary significantly in severity. Obesity emerged as a significant predictive factor for severe OSA [25]. Lastly, Santapuram et al. examined the association between autism severity, age at diagnosis, and factors like BMI in the context of OSA [26]. While the initial association between autism severity and age at OSA diagnosis did not prove to be statistically significant when accounting for other factors, both BMI and age at autism diagnosis appear to contribute to age at OSA diagnosis independently.

All studies, except one [23], are observational [20,21,22,24,25,26]. One of them is an RCT that comprises three different studies. Still, it has limitations, including slightly different study protocols, variations in the inclusion and exclusion criteria, and uncertainty about the presence of control groups [23]. The overall quality of these studies appears to be low.

## 4. Discussion

**Figure 2:**
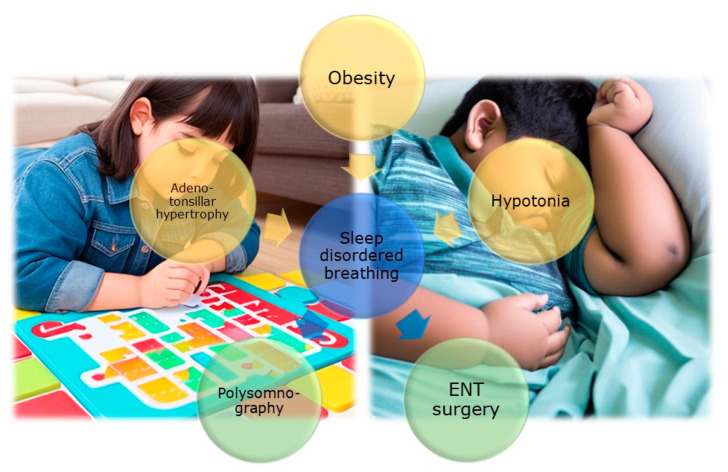
The figure (AI image generator https://dream.ai/create (accessed on 29 August 2023)) illustrates the association between autism and SDB in children. Key risk factors such as muscle hypotonia, obesity, and adenotonsillar hypertrophy are highlighted. Additionally, the figure underscores the importance of PSG in recognizing and treating SDB in autistic children. Lastly, the significance of otorhinolaryngological surgical intervention as an effective therapeutic option to alleviate symptoms of SDB and enhance the quality of life for children with autism is emphasized.

Research on the effects of SDB or OSA in children with ASD has revealed significant insights (Figure 2). One study demonstrated that 34% of children with ASD (*n* = 53, age 7.5 [4.8, 12.8] years) were diagnosed with OSA through PSG [20]. Another study reported that children with ASD (age 4.7 ± 1.14 years) who snore occasionally account for 25.4%, and 5.1% snore constantly. Children experiencing sleep apnea occasionally account for 3.5%, and those experiencing it frequently represent 0.6%, according to the CSHQ [23]. An elevated susceptibility to the development of sleep disorders, encompassing OSA, has been documented. The risk of sleep disorders in autistic children is increased by 96% compared to non-autistic children. Furthermore, children with ASD are also at a higher risk of undergoing PSG (increased by 274% compared to the control group) and ENT surgery (increased by 50% compared to the control group) [22]. Among 45 children with ASD (age 6.1 ± 2.8 years), 58% had OSA diagnosed through PSG, and 33% were obese [25]. In summary, these studies bring attention to the health issue of the elevated occurrence of SDB in children with ASD. The rising prevalence of children with ASD undergoing PSG underscores the growing demand for diagnostic and therapeutic interventions in this population [24] and exceptional attention to weight management [25]. Treatments for these disorders may encompass behavioral therapies, surgical interventions [22], medications, or a combination of these approaches.

The incidence of obesity in children with ASD is at least as high as, or even higher than, in the general population of children. Risk factors for a high BMI are advanced child age, high maternal BMI, low physical activity, and an increased likelihood of food selectivity [27]. Obesity has been identified as a predictive factor for SDB (OR 1.04, 95% CI 1.0–1.08, *p* = 0.02), especially for severe OSA, in children with ASD [25]. Moreover, the risk and rate of obesity in pediatric individuals with ASD are elevated [28], with causes primarily being attributed to sedentary lifestyles and improper dietary habits [29]. A study found that ferritin levels and BMI in children with ASD (*n* = 53, age 7.5 [IQR 4.8, 12.8] years) were not significantly correlated with OSA, suggesting the presence of other influential factors [20]. Another study indicated that BMI and age of autism diagnosis could independently impact the generation of OSA diagnosis [26].

The treatment of OSA (diagnosed through PSG) with A&T has proven to be essential in enhancing behavioral outcomes, irrespective of the child’s obesity and age [24]. In general, there have been reports of behavioral and cognitive improvements following A&T therapy in pediatric OSA, with authors consistently observing significant score enhancements in nearly all studies [30]. It is essential to conduct in-depth investigations to examine the advantages of A&T treatment in pediatric patients who have both ASD and OS.

OSA negatively impacts behaviors and overall well-being in children with ASD [24]. A study found a correlation between the number of pain-related behaviors exhibited in the previous week. It increased overall sleep-related problems, specifically shorter sleep duration, parasomnias (sleepwalking or nightmares), and SDB [21]. Some researchers have concluded that children with ASD are more likely to receive a diagnosis of sleep disorders, including SDB, and are more inclined to undergo related diagnostic and surgical procedures than individuals without ASD as controls [22].

Investigations are underway to explore the potential connection between connective tissue irregularities and ASD. However, the precise relationship between these two factors remains incompletely understood. Altered connectivity can give rise to heightened sensitivity to environmental stimuli. In ASD, there is a higher prevalence of asthma and allergic rhinitis, with a ratio of 5:1 compared to healthy subjects [31]. Frequent episodes of interstitial inflammation, immune-mediated forms of allergic asthma, bronchial hyperreactivity, nasal secretion, and a sensation of nasal obstruction have been observed following exposure to environmental allergens [32,33]. Very young children with common ear and upper respiratory symptoms appear to have an elevated risk of subsequently receiving an autism diagnosis or exhibiting high levels of autism-related traits [34]. However, it is important to note that a straightforward linear correlation between the degree of nasal obstruction and the severity of SDB is not consistently evident. In most cases of moderate or severe OSA, nasal obstruction is not the primary underlying factor [35].

Joint hypermobility has quite a high prevalence in ASD, so authors tend to include autism among hypermobility spectrum disorders (HSDs) [33]. Recognizing the differences between muscle weakness (hypotonia), tendon laxity, and joint hypermobility is complex, especially when dealing with individuals with autism, where it becomes an important issue [33,36]. However, hypotonia/joint hypermobility is also a recognizable marker of ASD [37]. Hypotonia/ligament laxity is when children have very “soft” or flaccid muscle tone [33]. The hypotonia/joint hypermobility observed was classified as mild to moderate and exhibited a widespread distribution across the entire body. Hypotonia was the most common motor symptom in a cohort of 154 children with ASD (51%), and it appears to improve over time. In the 2–6-year-old group, the prevalence of hypotonia was approximately 63% [36] at the age in which there is a high prevalence of SDB, mainly due to adenotonsillar hypertrophy [38,39]. In general, the atonia of skeletal muscles present during REM sleep might be exacerbated by the underlying hypotonia in children [40], and in ASD might increase the risk of OSA. When muscles are not adequately toned, the airways can become more collapsible. Identifying these joint-related issues paves the way for future research and the investigation of interventions designed to address joint hypermobility [33], hypotonia, and their effects on the respiratory health of children with ASD. It is important to emphasize that a customized physiotherapy approach can play a pivotal role in a comprehensive treatment plan for children with ASD who experience muscle hypotonia and are at risk of OSA.

This review has analyzed the relationship between ASD and SDB, particularly OSA, highlighting an intriguing landscape of complex correlations. Numerous research studies have established a notable correlation between ASD and the occurrence of OSA, with reported prevalence rates spanning from 34% to 58% [20,25]. Children diagnosed with ASD have also shown an elevated likelihood of developing sleep disorders, including OSA [22]. The risk and rate of obesity are increasing in pediatric individuals with ASD [25,28,29]. Obesity has been identified as a predictive factor for SDB, and BMI may be directly correlated with OSA in these children [20,26].

As obesity has been acknowledged as a contributing factor for SDB in pediatric individuals with ASD, it becomes essential to implement effective weight monitoring and management strategies tailored to this specific population. Addressing this concern may involve implementing lifestyle modification programs or targeted dietary interventions. While most participants in a study agreed that pediatricians should take the lead in managing obesity in children with ASD, only a few reported receiving adequate training for this role. As a result, they were more inclined to refer children with ASD to specialized services, such as dietitians or developmental–behavioral pediatricians [41]. Recognizing obesity as a predictive factor for SDB in children with ASD is crucial. Physicians should pay special attention to obese children with ASD because of their heightened risk of SDB, which can enable earlier diagnosis and treatment.

Children diagnosed with ASD might experience challenges in tolerating PSG [42,43] or discomfort sleeping in a different environment from their bed. However, some steps can help reduce anxiety during the PSG procedure, such as providing a detailed explanation of the procedure, identifying the child’s strengths, using accessories that reflect the child’s interests, and allowing family members to be present during the process to reduce anxiety, as well as paying close attention to the child’s needs. It is important to note that if a child cannot undergo PSG due to their condition, there are alternative methods for assessing SDB for the development of an appropriate therapeutic plan [43,44,45].

The review results have implications for practice, policy, and future research. OSA in children with ASD is complex, not related to BMI, necessitating consideration of various factors in treatment. Vocal behaviors and sleep quality impact SDB and should indicate sleep issues. Policies should encourage early sleep assessments and weight management strategies for timely OSA diagnosis and treatment in children with ASD. The treatment of SDB in children with ASD necessitates a multidisciplinary approach involving physicians, sleep therapists, nutritionists, and other professionals to ensure comprehensive and personalized treatment. Further research is necessary to understand the intricate causes of SDB in children with ASD and to develop effective interventions that consider the multifaceted nature of these causes and specific contributing factors.

## 5. Conclusions

In summary, this research illuminates the profound impact of SDB within the pediatric ASD population. The heightened prevalence of SDB underscores the imperative of a timely diagnosis and specialized therapeutic interventions. The presence of concurrent obesity necessitates a laser-focused approach to weight management strategies. While surgical interventions, such as A&T, aim to address OSA within the ASD context, a more expansive and rigorous research landscape is required to validate their efficacy definitively. The intricate interplay between ASD, SDB, and factors such as joint hypermobility beckons the application of multidisciplinary paradigms, potentially integrating the expertise of physiotherapists. Early sleep assessments and individualized weight management regimens emerge as pivotal components of this comprehensive care model. Concurrently, policy frameworks should be calibrated to support these initiatives. The quest for further research remains paramount, as it is the key to unravelling the multifarious etiological underpinnings of sleep disturbances in children with ASD and to formulating efficacious interventions.

## Figures and Tables

**Table 1:** This table summarizes the key points of various scientific studies available on the relationship between sleep disordered breathing (SDB) and autism spectrum disorder (ASD). Each column of the table provides specific information to enable an overall view of the characteristics and results of each study.

Primo Autore	Year of Publication	Design	Aim	Subjects	Methods	Results	Conclusions
Youssef J et al. [20]	2013	Retrospective chart review (Massachusetts)	To investigate the relationship between ferritin levels, fragmented sleep disorders, and joint movements in children with ASD.	Out of the 9,791 identified ASD children, 511 had ferritin level data, 377 had PSG data, and 53 had both ferritin and PSG data.	Review of ASD children’s records. PSG and ferritin analysis. Assessment of sleep fragmentation, limb movements. Comparison with the control group.	37% had sleep apnea. There was no significant difference in BMI or ferritin levels between ASD patients with or without OSA (*p* > 0.1). Ferritin levels did not predict abnormal sleep outcomes (*p* > 0.1).	No correlation between apnea, ferritin, and BMI.
Tudor, M.E et al. [21]	2015	[Longitudinal observational study] (USA)	Parental assessment. Correlation between pain and sleep issues.	Individuals with ASD (*n* = 62), child ages ranged from 3 to 18 years (9.39 ± 4.19 years).	NCCPC-R and CSHQ. Correlations between pain and sleep, including duration, parasomnias, and SDB. Impact of pain on sleep issues.	High scores in the SDB subscale were predicted by high scores in the Vocal subscale. SDB: mean subscale 3.99 ± 122; *n* = 35(56%) scoring > 0.	Sleep behaviors and vocalizations influence duration, parasomnias, and SDB.
Elrod MG et al. [22]	2016	Retrospective cohort study (Bethesda)	Risk assessment between ASD and controls for sleep disorders and diagnostic/surgical procedures.	48,762 children with ASD and controls (aged 2 to 18 years).	ASD (2000–2013). ASD matched 1:5 with controls for age, gender, and enrollment. Analysis of ICD-9 cm sleep disorders. RR and 95% CI were calculated using binary Poisson regression.	ASD children have a higher risk of sleep disorders, including OSA (RR: 1.97 [95% CI, 1.91–2.02]). Higher risk of PSG (RR: 3.74 [95% CI, 3.56–3.93]) and related surgeries (RR: 1.50 [95% CI, 1.46–1.54]).	Individuals with ASD have an elevated susceptibility to the emergence of sleep disorders, which includes OSA. They are more likely to have abnormal PSG results and undergo sleep-related surgeries than children without ASD.
Johnson CR et al. [23]	2016	Multisite RCT (Emory University, Indiana University, Ohio State University, University of Pittsburgh, University of Rochester, and Yale University)	Psychometric properties of the CSHQ in children with ASD.	310 children with ASD (age 4.7 ± 1.14 years)	The CSHQ (8 subscales): bedtime resistance, sleep onset delay, sleep duration, sleep anxiety, night wakings, parasomnias, SDB, and daytime sleepiness.	Loud, persistent snoring (5.1%), and other abnormal breathing behaviors (frequent apnea 0.6%) during sleep are relatively infrequent.	Loud snoring and other abnormal breathing behaviors (apneas) during sleep are rare.
Murata E et al. [24]	2017	Short-term retrospective study (Japan)	Behavioral changes after A&T for OSA in children with ASD.	*N* = 55 ASD children (*n* = 30 with OSA). Mean age: 7 years and 3 months (SD = 2 years and 5 months, range: 5–14 years) in the OSA group, and 7 years and 5 months (SD = 2 years and 0 months, range: 5–13 years) in the control group.	Children with untreated OSA and ASD control without OSA. OSA diagnosis: PSG, cardiorespiratory monitoring, oximetry. CBCL before and after treatment.	Pre-A&T scores for externalizing (*p* < 0.01), somatic problems (*p* < 0.05), anxiety/depression (*p* < 0.05), social issues (*p* < 0.01), thought problems (*p* < 0.01), delinquent behavior (*p* < 0.01), and aggressive behavior (*p* < 0.05) are significantly higher in the improved group compared to the no-change/deterioration group. Sex, A&T age, obesity indices, and severity of OSA based on AHI/3% ODI > 1 did not differ between the improved group and the no-change/deterioration group.	OSA in children with ASD should be treated regardless of obesity and age, even in cases of mild OSA, especially when more severe behavioral problems are present. We need to be aware of OSA in children with ASD.
Tomkies A et al. [25]	2019	Retrospective study (Texas)	Demographic and clinical characteristics, undergoing PSG, predictors of OSA and severe OSA.	45 children (age range 2–18 years, mean age 6.1 years).	PSG on children (born between 2009 and February 2015). Excluding severe comorbidities, tonsillectomy, and missing data. Collected age, sex, race, and clinical data. Analysis of OSA predictors.	The mean oAHI in children with OSA was 13.1 ± 18/h. 58% had OSA (AHI >1). 33% were obese (BMI ≥ 95th percentile). Severe OSA is significantly associated with weight (OR 1.0, 95% CI 1.0–1.1, *p* = 0.05). The mean AHI is 7.7/hour. 20% had severe OSA (AHI ≥ 10/h). There were no significant predictors for OSA except weight increase for severe OSA.	OSA is quite common in children, with considerable variability in severity. Obesity is associated with greater OSA severity. Weight appears to be a predictive factor for severe OSA.
Santapuram P et al. [26]	2022	Retrospective cohort study (USA)	A study comparing symptoms and age of OSA diagnosis. Children with and without ASD. Assessment of symptoms and age of OSA diagnosis. Identification of differences between groups.	Children with and without ASD. 166 children. The control group comprised 91 patients (54.9% male) with typical development and OSA. Age at OSA diagnosis: ASD 72.8 (45.6) months; control 73.4 (47.4) months, *p* = 0.999.	Review of clinical records for OSA (2019–2021). Analysis of diagnosis and treatment. Included children with OSA and A&T.	Less severity of autism was associated with a later age at OSA diagnosis (*p* < 0.001). Multivariate regression analysis did not reach statistical significance (*p* = 0.079). BMI and age at ASD diagnosis were independently associated with age at OSA diagnosis (*p* = 0.033 and *p* < 0.001, respectively).	Association between autism severity and age at OSA diagnosis. The association might not be significant when considering other factors simultaneously, such as BMI and age at ASD diagnosis. BMI and age at ASD diagnosis appear to have independent impacts on age at OSA diagnosis.

Legend: AHI, apnea–hypopnea index; ASD, autism spectrum disorder; A&T, adenotonsillectomy; BMI, body mass index; CSHQ, Children’s Sleep Habits Questionnaire; OSA, obstructive sleep apnea; PSG, polysomnography; SDB, sleep-disordered breathing.

## Data Availability

Data sharing not applicable.
